# Association between allergic rhinitis and metabolic conditions: a nationwide survey in Korea

**DOI:** 10.1186/s13223-015-0108-7

**Published:** 2016-01-22

**Authors:** In Cheol Hwang, Yong Joo Lee, Hong Yup Ahn, Sang Min Lee

**Affiliations:** Department of Family Medicine, Gachon University Gil Medical Center, Incheon, Republic of Korea; Department of Family Medicine, Seoul St. Mary’s Hospital, The Catholic University College of Medicine, 222 Banpo-daero, Seocho-gu, Seoul, 137-701 Republic of Korea; Department of Statistics, Dongguk University, Seoul, Republic of Korea; Division of Allergy, Department of Internal Medicine, Gil Medical Center, Incheon, Republic of Korea

**Keywords:** Allergic rhinitis, Cardiovascular risks, Metabolic syndrome, Impaired fasting glucose

## Abstract

**Background:**

Accumulating evidence indicates a strong correlation between allergic disease and cardiovascular risks. In spite of this, the data concerning the association between allergic rhinitis (AR) and cardiovascular risks is sparse and conflicting. This study aimed to investigate the association between AR prevalence and metabolic syndrome (MetS) in a large-scale, population-based survey, while considering the relevant risk factors.

**Methods:**

A nationwide cross-sectional study was conducted based on data from 30,590 subjects aged 19 years and older, from the Korean National Health and Nutrition Survey 2007–2013. The odds ratios (ORs) and 95 % confidence intervals (CIs) of AR prevalence, based on MetS status and the presence of any MetS component, were calculated using multiple logistic regression analyses.

**Results:**

Regarding the characteristics of patients with AR and/or MetS, some variables had significant associations with disease in inverse directions for AR and MetS. Multivariate logistic analysis, with adjustments for demographic variables and health habits, indicated that AR prevalence was significantly lower in subjects with MetS (OR 0.84; 95 % CI 0.76–0.93), high blood pressure (OR 0.85; 95 % CI 0.77–0.94), or impaired fasting glucose (OR 0.81; 95 % CI 0.73–0.89). Furthermore, high blood pressure and impaired fasting glucose were significant predictors for reduced AR prevalence, independently of other MetS components.

**Conclusion:**

In this population, AR was diagnosed less frequently in subjects with metabolic conditions. Well-designed prospective studies allowing for medical service utilization and collaborative basic research are warranted to elucidate the mechanism responsible for this inverse relationship.

## Background

Extensive studies have revealed that asthma patients are at greater risk of cardiovascular disease (CVD), with multiple mechanisms proposed [1]. Allergic rhinitis (AR) is believed to be an intermediate state between the normal healthy condition and clinical asthma [2], and it has been shown that individuals with AR are at greater risk of developing asthma [3]. AR has been suggested to be associated with CVD risks in a similar fashion [4]. The basis for this potential association is that individuals with significant AR symptoms are more likely to be habitual snorers, which then leads to higher blood pressure (BP). But, the findings of previous studies examining BP of AR patients are conflicting [5, 6]. Likewise, inconsistent results have been obtained with regard to the influence of AR history on CVD. In a prospective cohort study, participants with a history of hay fever had an adjusted hazard ratio of 1.87 for a stroke versus those without hay fever [7]. However, a recent nested case–control study demonstrated that AR patients were at lower risk for CVD [8].

The differing age of onset of AR and CVD, may complicate the relationship between them. The prevalence of AR is highest in the second to fourth decades of life, and then gradually diminishes; this is in contrast to the prevalence of CVD, which increases later in life [9]. It is also well known that AR occurs frequently in subjects of higher socioeconomic class [10]. To reduce the fragmentation found in previous studies, we aimed to investigate the association between AR prevalence and metabolic syndrome (MetS) in this large-scale population-based study with a consideration of the relevant risk factors.

## Methods

### Study design and subject selection

This study was based on data obtained from the Korean National Health and Nutrition Examination Survey 2007–2013, which was a nationwide population-based survey conducted by the Korean Ministry of Health and Welfare. Participants were randomly selected through a stratified, multistage, probability-sampling design according to sampling units based on age group from household registries as well as economic status, sex, and geographic area [11]. This study was approved by the Institutional Review Board of Gachon University Gil Medical Center.

We identified 44,118 participants of 19 years of age or older. Of these, subjects who were diagnosed with overt CVD (stroke or myocardial infarction, *n* = 1192), other allergic diseases (atopic dermatitis or asthma, *n* = 2157) and those who had no available data on the two main variables (AR, *n* = 8072; MetS, *n* = 2107) were excluded. Thus, a dataset of 30,590 patients was used in the final analysis.

### Data processing

Information on demographic characteristics (age, sex, household income, residency, education level, occupation and marital status), health behaviors (physical activity, smoking history, and alcohol consumption), and history of disease as diagnosed by a physician (AR, hypertension, type 2 diabetes, and dyslipidemia) was collected during the health interview. Waist circumference was measured at the narrowest point between the lower border of the rib cage and the iliac crest. BP was measured from the right arm after the subject had rested for 5 min in a sitting position using a standard mercury sphygmomanometer (Baumanometer, USA). Blood samples were obtained after a 12-h overnight fast, and fasting plasma glucose, triglyceride, and high-density lipoprotein cholesterol levels were measured using a Hitachi 700-110 Chemistry Analyzer (Hitachi, Tokyo, Japan).

The definition for MetS and its components was based on the National Cholesterol Education Program Adult Treatment Panel III guidelines, and we used the ethnicity-specific values for waist circumference based on data from the Korean Society for the Study of Obesity [12]. MetS was thus defined by the presence of three or more of the following criteria: central obesity (waist circumference ≥90 cm for men and ≥80 cm for women); systolic BP ≥130 mmHg or diastolic BP ≥85 mmHg; fasting plasma glucose levels ≥100 mg/dL; triglyceride levels ≥150 mg/dL; and low high-density lipoprotein cholesterol levels (<40 mg/dL for men and <50 mg/dL for women). Subjects who reported taking antihypertensive, antidiabetic, or lipid-lowering medications were considered to satisfy the corresponding criterion.

Economic status was classified into “low” or “high” according to family size-adjusted mean monthly household income, which was calculated by dividing household income by the square root of the number of persons in the household. The region in which the subject lived was categorized as either urban or rural. The capital city, its surrounding metropolitan area, and other metropolitan cities were grouped as urban areas; other areas were considered rural. Educational level was categorized as “middle school or lower” and “high school or beyond”. Marital status was classified as “married” or “unmarried”; “unmarried” included “single” and “divorced/separated/widowed”. Occupations were reorganized into three groups: “unemployed”, “white-collar” or “blue-collar”.

Physical activity was categorized as “regular physical activity” when participants engaged in moderate intensity activity more than five times per week, or vigorous activity more than three times per week, or “other”. The subject was considered a “never-smoker” if the subject had never smoked a cigarette, or otherwise an “ever-smoker.” For alcohol consumption, subjects were considered “frequent drinkers” if they consumed more than seven drinks (men) or five drinks (women) in one sitting more than 2 days per week [13].

### Statistical analyses

All analyses were performed using the STATA SE 9.2 (STATA Corp., TX). Descriptive statistics were presented as percentages. Differences in characteristics depending on AR or MetS status were evaluated using a Chi square test. Odds ratios (ORs) of AR prevalence in subjects with metabolic conditions and their 95 % confidence intervals (CIs) were calculated using both unadjusted and adjusted logistic regression models. All statistical tests were two-tailed, and results with *P*-values less than 0.05 were considered statistically significant.

## Results

Table 1 presents the characteristics of the patients organized by AR and MetS status. The overall prevalences of AR and MetS were 11.7 and 30.2 %, respectively. Some characteristics showing a significant difference between the status of both AR and MetS, had an inverse effect in the two conditions. For instance, in contrast to MetS, subjects being younger or of higher socioeconomic status was more common in the AR group than in the non-AR group.

**Table 1:** Subject characteristics, organized by allergic rhinitis (AR) and metabolic syndrome (MetS) status

**%**	No AR (*n* = 27,014)	AR (*n* = 3576)	P*	No MetS (*n* = 21,349)	MetS (*n* = 9241)	P*
Age, years						
19–30	11.6	20.9	<0.001	17.2	2.4	<0.001
30–39	18.7	31.3		24.9	9.3	
40–49	20.1	21.1		21.9	16.4	
50–59	19.1	14.9		16.4	23.9	
60–69	17.0	7.8		11.0	27.3	
≥70	13.5	4.1		8.7	20.8	
Female	56.7	61.6	<0.001	57.1	57.7	0.300
High economic status	55.9	65.4	<0.001	61.1	47.6	<0.001
Urban residency	76.4	85.3	<0.001	79.1	73.7	<0.001
Employed	60.6	61.1	0.529	63.5	54.1	<0.001
White-collar employment	19.3	29.7	<0.001	23.4	13.8	<0.001
Blue-collar employment	40.6	30.8	<0.001	39.4	39.6	0.694
Married	84.7	91.2	<0.001	89.0	78.6	<0.001
High education (≥high school)	62.2	85.8	<0.001	73.2	44.9	<0.001
Ever smoker	41.1	37.5	<0.001	40.5	41.2	0.271
Frequent drinker	5.0	3.7	0.001	4.4	6.0	<0.001
Regular physical activity	22.2	22.5	0.723	23.0	20.5	<0.001

* From a Chi square test

The ORs for AR prevalence in subjects with MetS and its components are shown in Table 2. Multivariate logistic analysis (Model I) revealed that the prevalence of AR was significantly lower in subjects with MetS (OR 0.84; 95 % CI 0.76–0.93), high BP (OR 0.85; 95 % CI 0.77–0.94), or impaired fasting glucose (OR 0.81; 95 % CI 0.73–0.89). Among the metabolic components, high BP and impaired fasting glucose were independent predictors for reduced AR prevalence (Model II).

**Table 2:** Odds ratios and 95 % confidence intervals of the prevalence of allergic rhinitis in subjects with metabolic conditions

	Unadjusted	Model I	Model II
Metabolic syndrome	0.53 (0.48–0.58)**	0.84 (0.76–0.93)*	
Central obesity	0.69 (0.64–0.75)**	0.93 (0.85–1.02)	0.97 (0.89–1.07)
High blood pressure	0.51 (0.47–0.55)**	0.85 (0.77–0.94)*	0.88 (0.79–0.97)*
Impaired fasting glucose	0.54 (0.50–0.59)**	0.81 (0.73–0.89)**	0.83 (0.75–0.92)**
Hypertriglyceridemia	0.69 (0.64–0.75)**	0.96 (0.88–1.05)	1.01 (0.91–1.11)
Low HDL-C	0.79 (0.73–0.85)**	1.00 (0.92–1.09)	1.03 (0.94–1.13)

*HDL-C* high-density lipoprotein cholesterol, *Model I* adjusted for demographic (age, sex, economic status, residency, type of job, marital status, and educational level) and health behavior (smoking status, alcoholic intake, and physical activity), *Model II* adjusted for the variables in Model 1 plus the other 4 metabolic conditions

* P < 0.01, ** *P* < 0.001

## Discussion

As the mechanisms behind our findings remain unclear, our findings should be interpreted with caution. To the best of our knowledge, there is no evidence supporting the preventive effect of AR for CVD risks. Conversely, a growing body of evidence suggests that inflammatory mediators and cells involved in AR may be implicated in the process of atherosclerosis [14, 15]. Clinically, AR symptoms can also contribute to reduced sleep quality, which is associated with an increased risk of hypertension, type 2 diabetes, and obesity [16, 17].

It is reasonable to interpret our results to signify a decreased prevalence of AR in subjects with metabolic conditions. Medical service utilization by socioeconomic status may explain these findings [18]. As individuals in the higher socioeconomic class are more likely to maintain their health, their likelihood of developing CVD decreases [19]. On the other hand, they may be less likely to endure uncomfortable conditions that are not life-threatening (i.e. AR), thus they are more often diagnosed with AR. A recent study reported that milder AR correlates with a reduction in the risk of intracranial hemorrhage [20]. Mediating effects of medical utilization on the association with AR might be attenuated for central obesity due to the difficulty of managing the disease [21] and for dyslipidemia because it is likely to be clinically overlooked [22].

Additionally, the helper T cell paradigm suggests that type 1 and type 2 helper T cells reciprocally counteract each other [23], which may explain the preventive effects of impaired fasting glucose on AR risk in this study. Patients with type 1 diabetes are known to exhibit a lower prevalence of allergic disease [24]. A pilot study extended to type 2 diabetes demonstrated that higher levels of plasma glucose were associated with lower risk for AR [25], which is similar to our findings.

This study has several limitations. First, we relied on self-reporting of AR and also did not differentiate between AR subtypes. Second, the cross-sectional design does not permit us to infer causality between AR prevalence and CVD components. To determine causality, a clinical trial in which the incidence of AR between two groups (for example, one group with and one without impaired fasting glucose) is compared over time, would be required.

## Conclusions

The present study is the largest to date to examine the relationship between AR prevalence and CVD risks. Our results underscore a significantly lower prevalence of AR in subjects with an identified metabolic dysfunction. Additional experimental and in-depth prospective studies are required to elucidate the mechanisms linking AR and CVD.

## Abbreviations

AR: allergic rhinitis
BP: blood pressure
CI: confidence interval
CVD: cardiovascular disease
MetS: metabolic syndrome
OR: odds ratio
